# Psychological pathways from daily discrimination to pain: a longitudinal mediation model

**DOI:** 10.1007/s10865-026-00670-6

**Published:** 2026-08-11

**Authors:** Nguyen P. Nguyen, Wajiha Masroor, Nnenna Uche, Jayoon Choi

**Affiliations:** 1 Department of Educational Psychology, Texas A&M University, College Station, USA; 2 Texas A&M University, College Station, USA

**Keywords:** Chronic pain, Daily discrimination, Self-esteem, Self-acceptance, Pain interference

## Abstract

Perceived discrimination, self-esteem, and self-acceptance have each been linked to pain outcomes, yet few studies have examined these factors together or investigated the psychological pathways connecting them. Grounded in the Biopsychosocial framework, this study explored the longitudinal mediating roles of self-esteem and self-acceptance in the relationship between perceived daily discrimination and pain interference. Archival data were drawn from the Midlife in the United States (MIDUS) study, with MIDUS II (2004–2006) as Time 1 and MIDUS III (2013–2014) as Time 2. The sample included 501 participants (ages 34–83) who self-reported chronic pain at both timepoints; the majority identified as White (92.2%) and female (60.7%). A multiple longitudinal mediation analysis revealed that perceived daily discrimination at Time 1 predicted greater pain interference at Time 2, an association mediated by lower levels of self-acceptance and self-esteem at Time 2. Notably, self-acceptance emerged as a more robust mediator than self-esteem. These findings suggest that the long-term impact of discrimination on pain outcomes may operate through its erosion of the sense of self, particularly self-acceptance. Clinically, these findings highlight the importance of addressing discrimination’s influence on self-acceptance as a critical pathway for intervention, prevention, and future research.

## Introduction

According to the Centers for Disease Control and Prevention, one in five Americans lives with chronic pain (Lucas & Sohi, 2024). While historically framed as a purely biomedical issue, growing research supports a more holistic understanding of pain, highlighting a bidirectional relationship where social and psychological stressors can precede, exacerbate, and prolong chronic physical pain (Hobson et al., 2022). This perspective aligns with the biopsychosocial model of chronic pain (Brown et al., 2018; Gatchel et al., 2012), positing that chronic pain is shaped by physiological, psychological, and social factors. Within this framework, discrimination has emerged as a prevalent social stressor that predicts unfavorable pain-related outcomes. Discrimination has been conceptualized as a form of social pain, a type of suffering that mirrors physical pain in its neural and emotional impact (Zhang et al., 2019). Not only does social pain worsen physical pain, but it also erodes psychological resources, resulting in lower self-esteem, reduced self-acceptance, and internalized distress (Pascoe & Richman, 2009). These psychological wounds, in turn, were found to predict more severe chronic pain symptoms and diminish overall physical health (Gatchel et al., 2012). Despite compelling evidence linking social pain and psychological processes to physical suffering, few studies have examined these factors together, particularly among midlife adults with chronic pain. To address this gap, the current study investigates the longitudinal mediation between perceived daily discrimination, self-appraisals (e.g., self-esteem and self-acceptance), and pain interference among midlife adults living with chronic pain.

### Theoretical framework: discrimination, self-appraisal, and pain interference

Perceived daily discrimination refers to the ongoing experience of being devalued or mistreated based on one’s social identity through everyday interactions embedded within broader sociocultural and historical contexts (Harnois, 2022). Within the biopsychosocial framework (Gatchel et al., 2012), discrimination represents a chronic social stressor that can become biologically and psychologically embodied over time, shaping long-term health and pain-related functioning. The Discrimination-Health Pathway Model (Pascoe & Richman, 2009) echoes this perspective, positing that discrimination adversely affects physical and mental health through multiple pathways, including psychological mechanisms such as self-appraisal. Particularly, repeated exposure to devaluation and social exclusion can erode a person’s sense of worth and self-regard (Duran et al., 2021). This, in turn, may contribute to heightened physiological arousal, increased allostatic load, and persistent hypervigilance, processes that have been linked to poorer chronic pain outcomes, including pain interference (Hobson et al., 2022; Ong et al., 2021; Pascoe & Richman, 2009).

Consistent with this conceptualization, emerging work suggests that discrimination can be understood as a form of social pain, with neural and affective consequences that overlap with physical pain processes (Hobson et al., 2022; Zhang et al., 2019). When social belonging is threatened, discrimination may activate multiple biological stress systems, including the sympathetic nervous system, the hypothalamic–pituitary–adrenal (HPA) axis, and related neuroimmune pathways (Hobson et al., 2022). Chronic activation of these systems has been associated with emotional dysregulation, heightened pain sensitivity, and reduced pain tolerance (Hobson et al., 2022; Renna, 2021). In pain research, one particularly relevant outcome is pain interference, defined as the extent to which pain disrupts daily functioning across domains such as sleep, mood, activity, and relationships (Cleeland & Ryan, 1994). Ong et al. (2021) found that daily discrimination was associated with greater pain interference among adults with chronic pain, supporting the possibility that discrimination may contribute to pain-related impairment through psychological mechanisms that shape how pain is experienced and managed over time. Taken together, extant literature suggests that perceived daily discrimination may predict worsened pain interference, in part through self-appraisal processes.

### Self-esteem as a mediator

Drawing from Pascoe and Richman’s (2009) model, self-esteem emerges as a psychological mechanism that may mediate the relationship between perceived daily discrimination and physical health outcomes. Conceptually, self-esteem is a core facet of self-appraisal, often defined as a person’s overall evaluative judgment of their own worth (Bailey, 2003). Self-esteem is dynamic and context-sensitive, and it is shaped by environmental conditions, achievements, and social experiences (Kim et al., 2023). Thus, self-esteem is vulnerable to disruption by negative social experiences, particularly those involving devaluation, rejection, or stigmatization (Kim et al., 2023).

The Modified Labeling Theory (MLT; Link et al., 1989) posits that the perceptions of negative social evaluation can lead individuals to adopt maladaptive internalized beliefs that undermine their self-appraisal (Waugh et al., 2014). Empirical studies also support this pathway. Within chronic pain populations, Hegarty and Wall (2013) found that stigmatizing and discriminatory behaviors predicted lowered self-esteem through internalized negative self-perceptions. Other studies also found that self-esteem significantly mediated the association between perceived discrimination and mental health outcomes, such as depression (Kim et al., 2023; Singh & Bhattacharyya, 2023). In the context of health and pain psychology research, lower self-esteem was associated with greater pain severity and interference, inflammatory conditions, and overall poorer psychological well-being (Hegarty & Wall, 2013; Juth et al., 2008; Wojtyna et al., 2024). Collectively, these findings highlight self-esteem as a psychological factor through which daily discrimination may predict worsened pain outcomes. In other words, self-esteem may mediate the association between perceived daily discrimination and pain outcomes, such as pain interference.“

### Self-acceptance as a mediator

Grounded in the Discrimination-Health Pathway Model (Pascoe & Richman, 2009), self-acceptance is another vital dimension of self-appraisal with growing relevance to health and pain-related outcomes. While literature recognizes a strong correlation between self-esteem and self-acceptance, these constructs are conceptually distinct. Self-esteem typically reflects a contingent evaluation of self-worth, often influenced by achievement, competence, and external validation; whereas self-acceptance denotes a broader, more unconditional positive regard for the self (MacInnes, 2006; Su et al., 2019). According to Su et al. (2019), self-acceptance involves holding a positive attitude toward oneself, including both strengths and flaws. As such, self-acceptance conceptually represents a more stable self-appraisal, reflecting an enduring capacity to embrace the self, regardless of achievements or external validation (MacInnes, 2006).

While self-acceptance may not be significantly dependent on external achievement, emerging evidence suggests that perceived discrimination can undermine self-acceptance, particularly by reinforcing internalized stigma and self-devaluation (Camp et al., 2020). In other words, negative societal messages may shape one’s internal dialogue, reinforcing self-doubt and reducing one’s ability to maintain a compassionate and stable self-acceptance (Elizur & Mintzer, 2001). Empirical evidence has shown that internalized negative societal messages might impede the development of self-acceptance among marginalized communities, and how self-acceptance could mediate the relationship between discrimination and psychological distress (Camp et al., 2020; Woodford et al., 2014).

In the context of pain psychology, acceptance-based constructs have long been explored as a resilience factor that promotes psychological flexibility, enhances coping, and reduces the subjective burden of pain (Åkerblom et al., 2015). Karayannis et al. (2023) also reported that individuals with a lower baseline of acceptance experienced the greatest increase in pain interference over time. Importantly, Martel et al. (2017) found that acceptance mediated the relationship between perceived injustice and pain outcomes (e.g., pain interference). These findings suggest that exposure to discrimination may hinder self-acceptance, which in turn may predict worsened pain-related outcomes.

### The current study

**Fig. 1:**
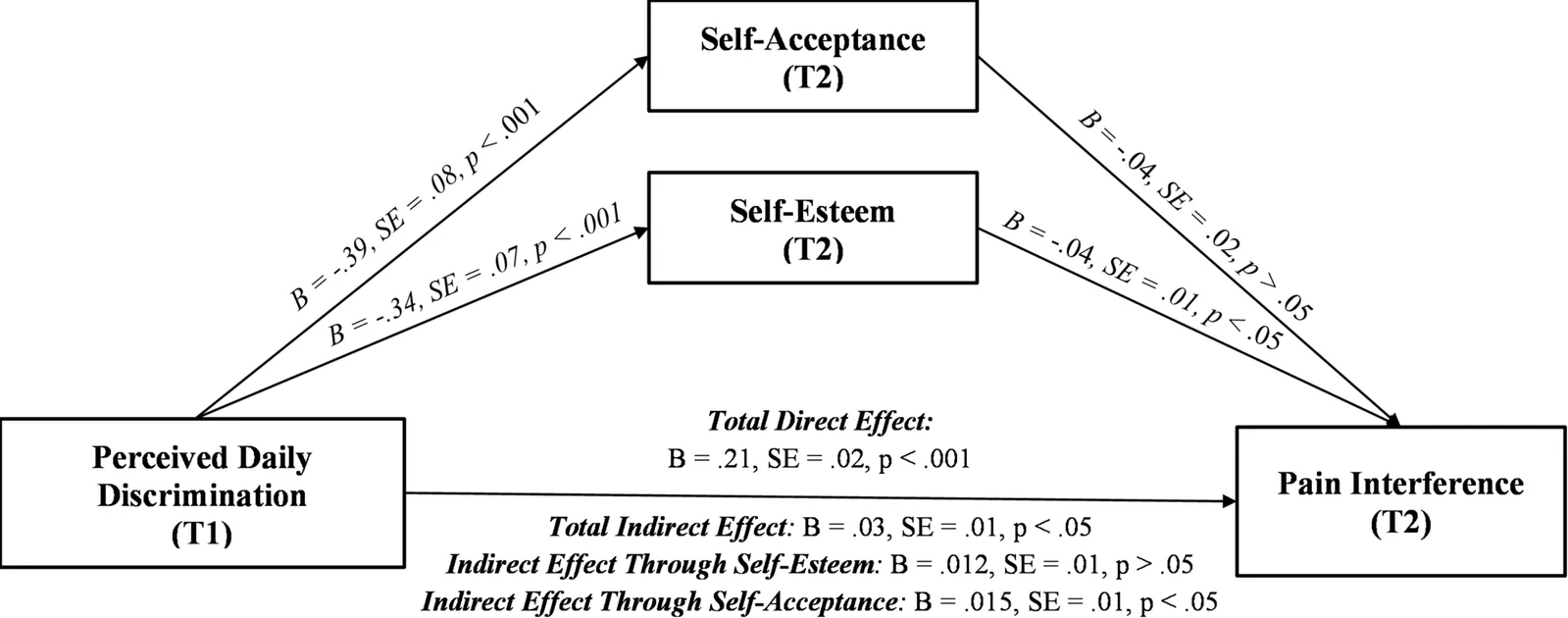
Multiple mediation model for perceived daily discrimination at time 1 (T1), self-esteem at time 2 (T2), self-acceptance at T2, and pain interference at T2, while controlling for gender, race/ethnicity, and pain interference at T1

Based on multiple theoretical frameworks and empirical research, self-esteem and self-acceptance have emerged as key psychological factors that may mediate the mechanisms through which perceived daily discrimination predicts pain-related outcomes. Despite their relevance, few studies have examined these constructs concurrently within the same models, particularly among midlife adults, a population at elevated risk for both chronic pain and cumulative experiences of discrimination. To advance the growing biopsychosocial approach to pain research and treatment, the present study proposed a longitudinal mediating model, examining whether perceived daily discrimination at Time 1 (T1) predicted higher pain interference at Time 2 (T2), through the mediating roles of self-esteem and self-acceptance at T2 (see Fig. 1).

## Methods

### Participants

The current study used the two waves of the National Longitudinal Study of Midlife in the United States (MIDUS). MIDUS II (Ryff et al., 2004–2006) represented T1, and MIDUS III (Ryff et al., 2013–2014) served as T2. There were 9–10 years between T1 and T2. The MIDUS III dataset only includes participants who completed measures at both time points. Participants were recruited using telephone banks. After consenting to the study, they completed the study by responding to the phone interview and mail-in survey.

There were 4,963 participants in MIDUS II and 3294 participants in MIDUS III. The retention rate after adjusting for mortality and ineligibility was 77% (Radler, 2014). Of the 3294 participants of both waves, 501 participants met the criteria for the current study. The criteria for the study were those who self-reported chronic pain (i.e., experiencing pain for longer than 3 months) at both timepoints. Participants’ ages ranged from 34 to 83 (M = 56.47, SD = 10.66) and 43–92 (M = 65.54, SD = 10.66) for T1 and T2, respectively. Regarding gender, 60.7% of participants identified as female and 39.3% identified as male. Furthermore, 462 participants self-identified as White (92.2%), 18 as Black (3.6%), 9 as Native American (1.8%), and 12 (2.4%) preferred not to answer. Regarding marital status, 349 participants at T1 and 310 participants at T2 were married or cohabited. Concerning sexual orientation, 471 participants identified as heterosexual (94%), 10 as homosexual (2%), 5 as bisexual (1%), and 15 participants refused to answer (3%).

### Measures

#### Perceived daily discrimination

Perceived daily discrimination was assessed using a 9-item measure of everyday discrimination developed by Williams et al. (1997). Items assess the frequency of routine unfair treatment (e.g., “You are treated with less respect than other people,” “You receive poorer service than other people at restaurants or stores”). Items were assessed on a 4-point Likert scale (1 = Often, 2 = Sometimes, 3 = Rarely, 4 = Never). Total scores were reverse-coded and summed, with higher scores indicating higher levels of perceived daily discrimination. Prior work using MIDUS has documented strong internal consistency and associations with mental health outcomes for this measure (Kessler et al., 1999). In the present study, internal consistency was high (Cronbach’s α = .0.92).

#### Self-esteem

Self-Esteem was measured using 7 items from the Rosenberg Self-Esteem Scale (Rosenberg, 1965). Using a 7-point Likert scale ranging from 1 (strongly disagree) to 7 (strongly agree), participants responded to statements such as "I take a positive attitude towards myself," "I am able to do things as well as most people," "On the whole, I am satisfied with myself." Some items were reverse coded, such as "At times I feel that I am no good at all" or "I wish I could have more respect for myself." Total scores were summed, with higher scores indicating higher levels of self-esteem. Previous studies found that self-esteem was positively associated with emotional regulation in coping associated with chronic pain (α = 0.85; Dysvik et al., 2005). For this study, the items yielded a Cronbach's alpha of 0.75.

#### Self-acceptance

Self-acceptance was measured using a 7-item subscale of self-acceptance in the Psychological Well-Being scale, used in the MIDUS study (Ryff & Singer, 1996). Items included: "When I look at the story of my life, I am pleased with how things have turned out", "In general, I feel confident and positive about myself", "I like most parts of my personality", and "When I compare myself to friends and acquaintances, it makes me feel good about who I am." Negatively worded items were reverse-coded, such as: "I feel like many of the people I know have gotten more out of life than I have", "In many ways I feel disappointed about my achievements in life", "My attitude about myself is probably not as positive as most people feel about themselves". Items were assessed on a 7-point scale (1 = strongly disagree to 7 = strongly agree) and summed into a scale score so that higher scores reflect higher levels of self-acceptance. In a previous study, the scale yielded a Cronbach’s alpha of 0.70, with self-acceptance being negatively associated with depression, anxiety, and panic disorder symptoms (Sanghvi et al., 2023). In this study, the Cronbach’s alpha for this scale was 0.85.

#### Pain interference

Pain interference was measured using a 5-item version of the Brief Pain Inventory’s Interference subscale (BPI; Cleeland & Ryan, 1994). Pain interference was assessed how pain interferes with five domains of participants’ lives, including daily activity, sleep, mood, social relationships, and enjoyment of life during the past week (e.g., "How much did your pain interfere with your sleep?", "How much did pain interfere with your enjoyment of life?"). Participants assessed their levels of pain interference in each of the domains on an 11-point Likert scale (0 = not at all to 10 = completely). Total score was obtained by summing the responses, with higher scores reflecting greater pain interference. In a study using the MIDUS sample, the BPI yielded a Cronbach’s alpha of 0.92, with pain interference significantly correlated with daily discrimination (Ong et al., 2021). In this study, the scale’s Cronbach’s alpha was .91.

#### Covariates

Participants’ race, gender, and pain interference at Time 1 were included as covariates. Race and gender were included because they are closely tied to structural exposure to discrimination and to health-related outcomes (Homan, 2019; Williams & Mohammed, 2013), and therefore represent important potential confounds in the proposed model. Pain interference at T1 was included to account for baseline pain-related functioning and to reduce the likelihood that associations observed at T2 simply reflect stable individual differences or pre-existing pain interference. Although other sociodemographic indicators (e.g., education, income) may shape both discrimination and health, they were not included as covariates in the primary models because these factors can also reflect downstream consequences of structural inequities and discrimination. Adjusting for downstream variables may constitute overadjustment and can attenuate or obscure the association of interest (Schisterman et al., 2009).

### Statistical analyses

All analyses were conducted using SPSS Version 28 (IBM Corp., 2021). Bivariate correlations were computed using Pearson’s r. To test the hypothesized mediation model, we conducted a parallel multiple mediation analysis using PROCESS macro (Model 4; Hayes, 2017). Specifically, we examined whether self-esteem (M1) and self-acceptance (M2) at Time 2 (T2) mediated the relationship between perceived daily discrimination at Time 1 (T1) and pain interference at T2, controlling for race, gender, and baseline pain interference. Standard OLS diagnostics were used to evaluate key model assumptions (e.g., linearity, homoscedasticity, and influential observations). Indirect effects were tested using nonparametric bootstrapping with 5000 resamples. This approach is recommended for mediation because bootstrapping provides more appropriate inference for indirect effects without assuming normality of the indirect effect’s sampling distribution (Alfons et al., 2022). Indirect effects were considered statistically significant if the 95% confidence interval did not include zero. The study was not pre-registered. All data, analysis code, and results are publicly available at: https://osf.io/4qptk/overview?view_only=edd58746238348bfa7a54d1a708ae65c.

## Results

Correlation analyses are presented in Table 1. Specifically, perceived daily discrimination at Time 1 (T1) was negatively correlated with self-esteem and self-acceptance at Time 2 (T2), both with moderate effect sizes, and positively correlated with pain interference at T2 with a small effect size. Additionally, self-esteem and self-acceptance at T2 were both negatively associated with pain interference at T2, showing moderate effect sizes. All correlations were statistically significant at *p* < 0.001. The results revealed that perceived daily discrimination at T1 significantly predicted lower self-esteem at T2 (*B* =  − 0.34, *SE* = 0.07, *p* < 0.001) and lower self-acceptance at T2 (*B* =  − 0.39, *SE* = 0.08, *p* < 0.001). Self-esteem at T2 showed a non-significant negative association with pain interference at T2 (*B* =  − 0.04, *SE* = 0.02, *p* = 0.093), while self-acceptance at T2 was a significant negative predictor of pain interference at T2 (*B* =  − 0.04, *SE* = 0.01, *p* < 0.05).

**Table 1:** Correlation analyses for perceived daily discrimination at T1, self-esteem at T2, self-acceptance at T2, and pain-interference at T2, with *n* = 501

Correlation analyses						
Variable	M	SD	1	2	3	4
1. Perceived daily discrimination (T1)	31.5	4.8	–			
2. Self-esteem (T2)	36.25	7.64	− 0.31**	–		
3. Self-acceptance (T2)	19.78	8.68	− 0.30**	.77**	–	
4. Pain interference (T2)	18.15	13.29	0.21**	− 0.33**	− 0.33**	–

^**^ *p* < .001

The total effect of perceived daily discrimination at T1 on pain interference at T2 was significant (*B* = 0.21, *p* < 0.001). However, after accounting for the covariates (i.e., race, gender, baseline pain interference at T1) and the mediators (self-esteem and self-acceptance at T2), the direct effect of perceived daily discrimination at T1 on pain interference at T2 was no longer significant (*B* =  − 0.003, *SE* = 0.02, *p* = 0.900), suggesting that the association was significantly mediated. The total indirect effect of perceived daily discrimination at T1 on pain interference through self-esteem and self-acceptance at T2 was statistically significant (*B* = 0.03, *SE* = 0.01, 95% *CI* [0.01, 0.04]), supporting the hypothesized multiple mediation model. Among the two mediators, only self-acceptance at T2 demonstrated a statistically significant indirect effect (*B* = 0.015, *SE* = 0.01, 95% *CI* [0.0001, 0.03]), while the indirect effect through self-esteem did not reach significance (*B* = 0.012, *SE* = 0.01, 95% *CI* [− 0.002, 0.03]). The mediation model is presented in Fig. 1.

## Discussion

This study is among the first to concurrently examine the mediating roles of self-acceptance and self-esteem in the association between perceived daily discrimination and pain interference. The study’s findings highlight the long-term associations between perceived discrimination and self-appraisal, such that adults who reported higher levels of daily discrimination also reported significantly lower levels of self-esteem and self-acceptance nearly a decade later. These results aligned with previous theory and research, depicting the internalization process wherein individuals absorb and integrate discriminatory experiences into their self-perceptions (Link et al., 1989; Waugh et al., 2014). Furthermore, the study also illustrates the adverse association between perceived daily discrimination and pain interference. These findings reinforce the growing body of evidence on the overlapping nature of social and physical pain, highlighting how experiences of social pain (e.g., discrimination) can intensify physical pain, particularly among midlife adults with chronic pain (Nguyen & Kim, 2025; Nguyen et al., 2023).

Findings from the multiple mediation model offer novel insights into the potential psychological pathways linking perceived discrimination to later pain interference through self-esteem and self-acceptance. Prior research has consistently underscored the importance of self-esteem and self-acceptance in pain adjustment, including greater pain resilience and lower levels of pain intensity (Kalapurakkel et al., 2015). Building on this work, the present findings suggest that higher perceived discrimination was associated with lower self-esteem and self-acceptance, which in turn were predictive of higher pain interference. Consistent with previous literature and theoretical frameworks (Gatchel et al., 2012), these results highlight the relevance of psychological processes in understanding the way to which discrimination predicts pain-related functioning. Importantly, even though the proposed mediation model was theoretically guided (e.g., the Discrimination–Health Pathway Model; Pascoe & Richman, 2009) and baseline pain interference was included to account for prior pain-related functioning, the mediators and outcome were assessed at the same time point; thus, temporal ordering constructs cannot be established, and causal interpretation is not warranted. Thus, the findings are best interpreted as that self-esteem and self-acceptance may represent plausible pathways linking discrimination to pain-related functioning, rather than as definitive evidence of causal mediation.

When examined separately, only self-acceptance emerged as a significant mediator. While both self-esteem and self-acceptance demonstrated similar associations with pain interference in terms of effect size (Betas), bootstrapping analyses revealed that self-acceptance was a more reliable and consistent predictor of pain interference. Differences in the psychometric properties of the two scales may partially contribute to these differences. Firstly, the self-acceptance scale places greater emphasis on integrating life experiences holistically (e.g., “When I look at the story of my life, I am pleased with how things have turned out”), which may better depict long-term stability in self-perception. The Self-esteem scale may place heavier emphasis on self-respect and personal satisfaction (e.g., “I wish I could have more respect for myself”), making it potentially more susceptible to fluctuations based on recent experiences. Furthermore, the self-esteem scale contains items reflecting more extreme emotional reactions (e.g., “All in all, I am inclined to think that I am a failure”), which may contribute to greater volatility in self-perception. Meanwhile, the self-acceptance scale, while also including negative self-reflection items, frames them as less self-deprecating (e.g., “In many ways, I feel disappointed about my achievements in life”) and allows room for imperfection (e.g., “I like most parts of my personality”). Consistent with these differences, the internal consistency of the two scales also diverges. The Rosenberg Self-Esteem Scale demonstrated lower reliability (Cronbach’s α = 0.75), whereas self-acceptance exhibited higher reliability (Cronbach’s α = 0.85) in the current study, and similar trends have been observed in previous research using the MIDUS dataset (e.g., Stokes, 2020). This suggests that self-acceptance is a more coherent and internally stable construct, while self-esteem may be more fluctuating.

Conceptually, while self-acceptance and self-esteem both reflect positive self-appraisal and self-evaluation, they differ in fundamental ways (Popov, 2019). Self-esteem is often contingent on external validation, personal achievements, and social comparisons, making it inherently more volatile and susceptible to external influences (Popov, 2019; Shrauger & Lund, 1975). In contrast, self-acceptance represents a more stable, unconditional affirmation of oneself, encompassing both strengths and limitations (Ellis & Ellis, 2011). These distinctions also align with broader findings in psychological research. While interventions targeting both self-esteem and self-acceptance have been linked to positive health outcomes, some evidence suggests that increases in self-esteem may not necessarily contribute to long-term psychological adjustment or resilience (Popov, 2019; Yu & McCracken, 2025). Additionally, self-esteem has not been found to reliably predict relationship quality, prevent maladaptive behaviors, or enhance sustained well-being (Baumeister et al., 2003; Yu & McCracken, 2025). By contrast, self-acceptance has been more strongly associated with general psychological well-being, life satisfaction, and resilience (Macinnes, 2006; Popov, 2019). Taken together, these findings provided initial evidence that self-acceptance may serve as a more robust and reliable psychological factor in the mechanism of how daily discrimination adversely predicted pain interference.

It is important to note that the self-acceptance measure in the current study is conceptually distinct from “acceptance” constructs emphasized in the pain literature, particularly those grounded in Acceptance and Commitment Therapy (ACT). In ACT-oriented models, acceptance typically refers to willingness to experience internal events (e.g., pain, distressing thoughts) without excessive avoidance or control efforts (Feliu-Soler et al., 2018). By contrast, the measure used here more closely captures a global stance of self-regard and self-appraisal (i.e., self-evaluative acceptance) rather than experiential willingness. Accordingly, the findings are best interpreted as implicating self-appraisal acceptance, rather than experiential acceptance, as a plausible pathway linking discrimination to pain interference.

## Limitations and future directions

The study’s findings should be interpreted considering several important limitations. First, the measurement of self-acceptance was derived from a subscale of the Psychological Well-Being Scale, consisting of only seven items, which may not fully capture the multifaceted nature of self-acceptance. This limitation is inherent to studies utilizing archival datasets, where researchers must work within the constraints of available measures. Despite the scale demonstrating good internal validity, future research should employ more comprehensive and validated measures of self-acceptance and self-esteem. Furthermore, future studies should incorporate validated ACT-consistent measures (e.g., experiential avoidance or psychological flexibility) and pain acceptance instruments to test whether these acceptance-related constructs operate as parallel or sequential mechanisms alongside self-evaluative processes. Additionally, even though the use of pain interference as the primary pain outcome aligns well with the biopsychosocial framework of pain (Gatchel et al., 2012), it may not capture other important aspects of pain, such as pain intensity, duration, nature, or disability. Incorporating a more comprehensive assessment of pain in future research would help provide fuller assessments of participants’ pain outcomes.

Another limitation is that the mediators and the outcome were assessed concurrently at Wave 2, which limits confidence in the assumed directional ordering among these variables and temporal precedence cannot be definitively established. Hence, the indirect effects should be interpreted as theoretically consistent statistical pathways rather than causal mechanisms, and future work using three-wave or intensive longitudinal designs is needed. Furthermore, although the long interval between MIDUS II and MIDUS III is a strength for examining longer-term associations, it also limited the ability to account for intervening biological, psychological, and social changes across the approximately 10-year span. Participants may have experienced major life events, new medical diagnoses, injuries, changes in pain and functional capacity, relationship transitions, or shifts in socioeconomic circumstances that could influence self-appraisal and pain interference. Because MIDUS did not assess these factors repeatedly across the full interval, future studies should include more frequent follow-ups to better account for intervening diagnoses and life events that can impact self-appraisal, pain, and health over time.

Additionally, although this study highlights self-appraisal as one pathway from discrimination to pain interference, discrimination and pain are also shaped by other important social conditions that extend beyond the individual. Future work should examine key social and structural factors, such as education, income, social support, and relationship context, not simply as covariates to adjust, but as primary constructs that can either moderate or mediate the discrimination–health cascades. These factors may influence exposure to discrimination, the resources available to cope with it, and the extent to which discrimination undermines health over time. Testing these variables as predictors, moderators, mediators, and outcomes will provide a richer biopsychosocial account of when discrimination translates into pain-related impairment and where intervention efforts may be most effective.

Lastly, although MIDUS provides a valuable community-based sample, its limited representation of racial, ethnic, and sexual minority participants restricts the generalizability of these findings. Because exposure to discrimination is not evenly distributed across social groups, and is often more frequent, severe, and chronic among certain minoritized populations, results derived primarily from the current sample may not translate to groups who experience higher and qualitatively different forms of discrimination (Banaji & Fiske, 2021). This limited diversity may also restrict the range and variability of discrimination experiences in the sample, which could have weakened observed associations and limited the ability to detect group-specific pathways. Future studies should test these pathways in more diverse, intersectionally represented samples and use designs that can directly examine whether the mechanisms differ across groups. This would clarify for whom, and under what conditions, discrimination may shape self-appraisal and pain-related outcomes.

## Clinical implications

The present findings carry meaningful implications for both pain research and clinical practice. Consistent with the biopsychosocial model of pain (Gatchel et al., 2012), the results suggest that discrimination is not merely a background stressor, but a clinically relevant social exposure that can shape psychological functioning and, in turn, pain-related interference. In practice, clinicians can strengthen assessment and case formulation by routinely attending to clients’ lived experiences of discrimination and to the self-related processes that often follow, including shame, self-criticism, and difficulty with self-acceptance. This framing can also support patient education. For many individuals, particularly those from historically marginalized communities, naming discrimination as part of the pain context can demystify symptoms, validate distress, and increase adherence for integrative care that extends beyond biomedical approaches alone (Meint & Edwards, 2018).

Importantly, the present study suggests that self-acceptance may be a more consistent psychological predictor of pain interference than self-esteem when examined alongside one another. Rather than focusing only on bolstering self-esteem and confidence, the current findings point to the potential value of strengthening self-acceptance and reducing self-judgment as part of pain care, especially when discrimination exposure is salient. Approaches such as Acceptance and Commitment Therapy and DBT-informed may be well-suited to interrupt the discrimination–health cascades by strengthening psychological flexibility, emotion regulation, and radical acceptance, even in the presence of ongoing stress (Norman-Nott et al., 2025; Trindade et al., 2021). These efforts may be further strengthened when paired with attention to social resources. For instance, treatment planning can also include interventions that promote social support and belongingness, given evidence that social support and community resources can buffer the harmful effects of discrimination on self-processes and health outcomes (Pascoe & Richman, 2009).

Finally, cultivating self-acceptance should not be conflated with passive acceptance of injustice. A clinically responsible approach integrates self-acceptance with critical awareness and empowerment by helping clients name structural inequities, clarify personal agency, and practice concrete forms of self-advocacy and boundary-setting (Nguyen & Kim, 2025). These findings also underscore the role of clinicians and scientists in advancing more socially informed and equitable pain care (Mankelow et al., 2022). This includes reducing stigma in pain treatment, strengthening systems that recognize discrimination as a meaningful determinant of health, and investing in education for healthcare providers so that biopsychosocial formulations and referrals to integrated care are delivered with greater clarity and confidence (Mankelow et al., 2022; Ryan et al., 2024). In doing so, clinical work can move beyond symptom management toward interventions that validate lived experience, protect agency, and promote well-being in the context of structural adversity.
